# Segmentation of roots in soil with U-Net

**DOI:** 10.1186/s13007-020-0563-0

**Published:** 2020-02-08

**Authors:** Abraham George Smith, Jens Petersen, Raghavendra Selvan, Camilla Ruø Rasmussen

**Affiliations:** 1 Department of Plant and Environmental Sciences, University of Copenhagen, Højbakkegaard Allé 13, 2630 Taastrup, Denmark; 2 Department of Computer Science, University of Copenhagen, Universitetsparken 1, 2100 Copenhagen Ø, Denmark

**Keywords:** Roots, Convolutional neural network, Rhizotron, Deep learning, Phenotyping, Image analysis, Root intersection method

## Abstract

**Background:**

Plant root research can provide a way to attain stress-tolerant crops that produce greater yield in a diverse array of conditions. Phenotyping roots in soil is often challenging due to the roots being difficult to access and the use of time consuming manual methods. Rhizotrons allow visual inspection of root growth through transparent surfaces. Agronomists currently manually label photographs of roots obtained from rhizotrons using a line-intersect method to obtain root length density and rooting depth measurements which are essential for their experiments. We investigate the effectiveness of an automated image segmentation method based on the U-Net Convolutional Neural Network (CNN) architecture to enable such measurements. We design a data-set of 50 annotated chicory (*Cichorium intybus* L.) root images which we use to train, validate and test the system and compare against a baseline built using the Frangi vesselness filter. We obtain metrics using manual annotations and line-intersect counts.

**Results:**

Our results on the held out data show our proposed automated segmentation system to be a viable solution for detecting and quantifying roots. We evaluate our system using 867 images for which we have obtained line-intersect counts, attaining a Spearman rank correlation of 0.9748 and an $r^2$ of 0.9217. We also achieve an $F_1$ of 0.7 when comparing the automated segmentation to the manual annotations, with our automated segmentation system producing segmentations with higher quality than the manual annotations for large portions of the image.

**Conclusion:**

We have demonstrated the feasibility of a U-Net based CNN system for segmenting images of roots in soil and for replacing the manual line-intersect method. The success of our approach is also a demonstration of the feasibility of deep learning in practice for small research groups needing to create their own custom labelled dataset from scratch.

## Background

High-throughput phenotyping of roots in soil has been a long-wished-for goal for various research purposes [1–4]. The challenge of exposing the architecture of roots hidden in soil has promoted studies of roots in artificial growth media [5]. However, root growth is highly influenced by physical constraints [6] and such studies have shown to be unrepresentative of roots in soil [7, 8].

Traditionally studies of roots in soil have relied on destructive and laborious methods such as trenches in the field and soil coring followed by root washing [9]. Recently 3D methods such as X-ray computed tomography [10] and magnetic resonance imaging [11] have been introduced, but these methods require expensive equipment and only allow small samples.

Since the 1990, rhizotrons [12–14] and minirhizotrons [15, 16] which allow non-invasive monitoring of spatial and temporal variations in root growth in soil, have gained popularity. Minirhizotrons facilitate the repeated observation and photographing of roots through the transparent surfaces of below ground observation tubes [17].

A major bottleneck when using rhizotron methods is the extraction of relevant information from the captured images. Images have traditionally been annotated manually using the line-intersect method where the number of roots crossing a line in a grid is counted and correlated to total root length [18, 19] or normalised to the total length of grid line [20]. The line-intersect method was originally developed for washed roots but is now also used in rhizotron studies where a grid is either directly superimposed on the soil-rhizotron interface [21, 22] or indirectly on recorded images [23, 24]. The technique is arduous and has been reported to take 20 min per metre of grid line in minirhizotron studies [25]. Line-intersect counts are not a direct measurement of root length and do not provide any information on architectural root traits such as branching, diameter, tip count, growth speed or growth angle of laterals.

To overcome these issues, several attempts have been made to automate the detection and measurement of roots, but all of them require manual supervision, such as mouse clicks to detect objects [26, 27].

The widely used “RootFly” software provides both manual annotation and automatic root detection functionality [28]. Although the automatic detection worked well on the initial three datasets the authors found it did not transfer well to new soil types (personal communication with Stan Birchfield, September 27, 2018).

Following the same manual annotation procedure as in RootFly, [29] calculated that it takes 1–1.5 h per 100 cm^2^ to annotate images of roots from minirhizotrons, adding up to thousands of hours for many minirhizotron experiments. Although existing software is capable of attaining much of the desired information, the annotation time required is prohibitive and severely limits the use of such tools.

Image segmentation is the splitting of an image into different meaningful parts. A fully automatic root segmentation system would not just save agronomists time but could also provide more localized information on which roots have grown and by how much as well as root width and architecture.

The low contrast between roots and soil has been a challenge in previous attempts to automate root detection. Often only young unpigmented roots can be detected [30] or roots in black peat soil [31]. To enable detection of roots of all ages in heterogeneous field soils, attempts have been made to increase the contrast between soil and roots using custom spectroscopy. UV light can cause some living roots to fluoresce and thereby stand out more clearly [3] and light in the near–infrared spectrum can increase the contrast between roots and soil [32].

Other custom spectroscopy approaches have shown the potential to distinguish between living and dead roots [33, 34] and roots from different species [35, 36]. A disadvantage of such approaches is that they require more complex hardware which is often customized to a specific experimental setup. A method which works with ordinary RGB photographs would be attractive as it would not require modifications to existing camera and lighting setups, making it more broadly applicable to the wider root research community. Thus in this work we focus on solving the problem of segmenting roots from soil using a software driven approach.

Prior work on segmenting roots from soil in photographs has used feature extraction combined with traditional machine learning methods [37, 38]. A feature extractor is a function which transforms raw data into a suitable internal representation from which a learning subsystem can detect or classify patterns [39]. The process of manually designing a feature extractor is known as feature engineering. Effective feature engineering for plant phenotyping requires a practitioner with a broad skill-set as they must have sufficient knowledge of both image analysis, machine learning and plant physiology [40]. Not only is it difficult to find the optimal description of the data but the features found may limit the performance of the system to specific datasets [41]. With feature engineering approaches, domain knowledge is expressed in the feature extraction code so further programming is required to re-purpose the system to new datasets.

Deep learning is a machine learning approach, conditioned on the training procedure, where a machine fed with raw data automatically discovers a hierarchy of representations that can be useful for detection or classification tasks [39]. Convolutional Neural Networks (CNNs) are a class of deep learning architectures where the feature extraction mechanism is encoded in the weights (parameters) of the network, which can be updated without the need for manual programming by changing or adding to the training data. Via the training process a CNN is able to learn from examples, to approximate the labels or annotations for a given input. This makes the effectiveness of CNNs highly dependent on the quality and quantity of the annotations provided.

Deep learning facilitates a decoupling of plant physiology domain knowledge and machine learning technical expertise. A deep learning practitioner can focus on the selection and optimisation of a general purpose neural network architecture whilst root experts encode their domain knowledge into annotated data-sets created using image editing software.

CNNs have now established their dominance on almost all recognition and detection tasks [42–45]. They have also been used to segment roots from soil in X-ray tomography [46] and to identify the tips of wheat roots grown in germination paper growth pouches [41]. CNNs have an ability to transfer well from one task to another, requiring less training data for new tasks [47]. This gives us confidence that knowledge attained from training on images of roots in soil in one specific setup can be transferred to a new setup with a different soil, plant species or lighting setup.

The aim of this study is to develop an effective root segmentation system using a CNN. For semantic segmentation tasks CNN architectures composed of encoders and decoders are often used. These so-called encoder-decoder architectures firstly transform the input using an encoder into a representation with reduced spatial dimensions which may be useful for classification tasks but will lack local detail, then a decoder will up-sample the representation given by the encoder to a similar resolution as the original input, potentially outputting a label for each pixel. Another encoder-decoder based CNN system for root image analysis is RootNav 2.0 [48] which is targeted more towards experimental setups with the entire root system visible, where it enables extraction of detailed root system architecture measurements. We use the U-Net CNN encoder-decoder architecture [49], which has proven to be especially useful in contexts where attaining large amounts of manually annotated data is challenging, which is the case in biomedical or biology experiments.

As a baseline machine learning approach we used the Frangi vessel enhancement filter [50], which was originally developed to enhance vessel structures on images of human vasculature. Frangi filtering represents a more traditional and simpler off-the-shelf approach which typically has lower minimum hardware requirements when compared to U-Net.

We hypothesize that (1) U-Net will be able to effectively discriminate between roots and soil in RGB photographs, demonstrated by a strong positive correlation between root length density obtained from U-Net segmentations and root intensity obtained from the manual line-intersect method. And (2) U-Net will outperform a traditional machine learning approach with larger amounts of agreement between the U-Net segmentation output and the test set annotations.

## Methods

**Fig. 1:**
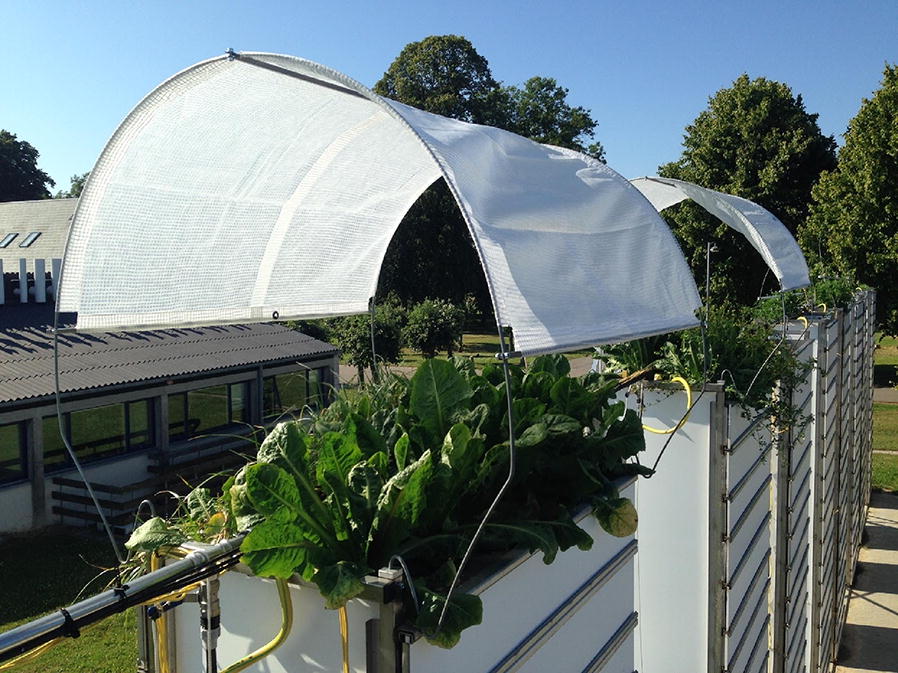
Chicory (*Cichorium intybus* L.) growing in the rhizotron facility

We used images of chicory (*Cichorium intybus* L.) taken during summer 2016 from a large 4 m deep rhizotron facility at University of Copenhagen, Taastrup, Denmark (Fig. 1). The images had been used in a previous study [51] where the analysis was performed using the manual line-intersect method. As we make no modifications to the hardware or photographic procedure, we are able to evaluate our method as a drop-in replacement to the manual line-intersect method.

The facility from which the images were captured consists of 12 rhizotrons. Each rhizotron is a soil filled rectangular box with 20 1.2 m wide vertically stacked transparent acrylic panels on two of its sides which are covered by 10 mm foamed PVC plates. These plates can be removed to allow inspection of root growth at the soil-rhizotron interface. There were a total of 3300 images which had been taken on 9 different dates during 2016. The photos were taken from depths between 0.3 and 4 m. Four photos were taken of each panel in order to cover its full width, with each individual image covering the full height and 1/4 of the width (For further details of the experiment and the facility see [51]). The image files were labelled according to the specific rhizotron, direction and panel they are taken from with the shallowest which is assigned the number 1 and the deepest panel being assigned the number 20.

Line-intersect counts were available for 892 images. They had been obtained using a version of the line-intersect method [18] which had been modified to use grid lines [19, 52] overlaid over an image to compute root intensity. Root intensity is the number of root intersections per metre of grid line in each panel [20].

In total four different grids were used. Coarser grids were used to save time when counting the upper panels with high root intensity and finer grids were used to ensure low variation in counts from the lower panels with low root intensity. The 4 grids used had squares of sizes 10, 20, 40 and 80 mm. The grid size for each depth was selected by the counter, aiming to have at least 50 intersections for all images obtained from that depth. For the deeper panels with less roots, it was not possible to obtain 50 intersections per panel so the finest grid (10 mm) was always used.

To enable comparison we only used photos that had been included in the analysis by the manual line-intersect method. Here photos containing large amounts of equipment were not deemed suitable for analysis. From the 3300 originals, images from panels 3, 6, 9, 12, 15 and 18 were excluded as they contained large amounts of equipment such as cables and ingrowth cores. Images from panel 1 were excluded as it was not fully covered with soil. Table 1 shows the number of images from each date, the number of images remaining after excluding panels unsuitable for analysis and if line-intersect counts were available.

**Table 1:** Number of images from each date

Date	Total images	Included	Line-intersect counts
21/06/16	192	168	Yes
27/06/16	296	180	No
04/07/16	320	196	Yes
11/07/16	348	216	No
18/07/16	396	248	Yes
25/07/16	420	268	No
22/08/16	440	280	Yes
05/09/16	440	276	No
21/09/16	448	280	No

Not all images are included as they may contain large amounts of equipment

**Fig. 2:**
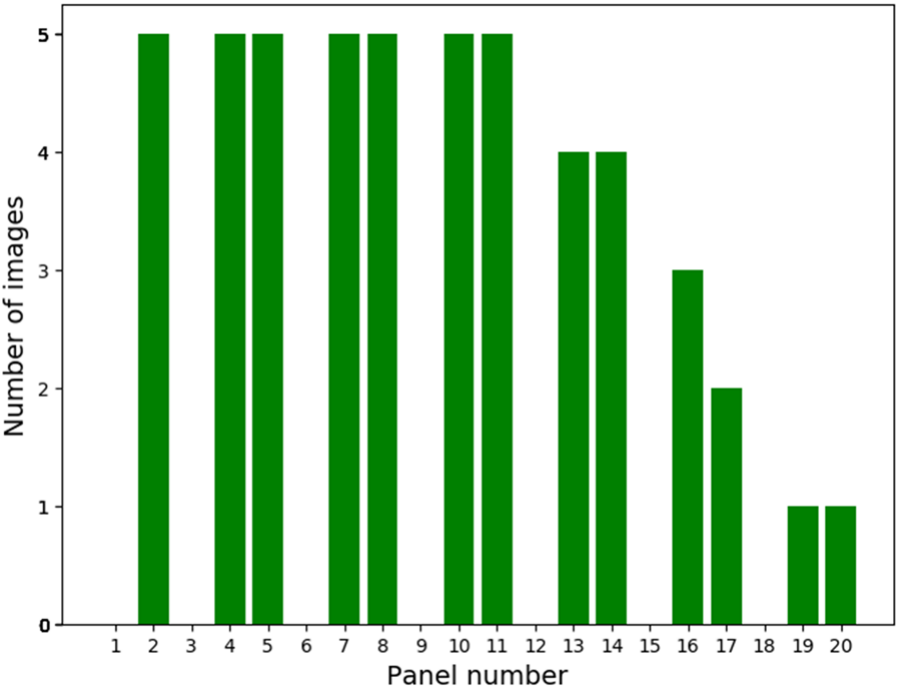
The number of images selected for annotation from each panel depth

Deeper panels were sometimes not photographed as when photographing the panels the photographer worked from the top to the bottom and stopped when it was clear that no deeper roots could be observed. We took the depth distribution of all images obtained from the rhizotrons in 2016 into account when selecting images for annotation in order to create a representative sample (Fig. 2). After calculating how many images to select from each depth the images were selected at random.

The first 15 images were an exception to this. They had been selected by the annotator whilst aiming to include all depths. We kept these images but ensured they were not used in the final evaluation of model performance as we were uncertain as to what biases had led to their selection.

### Annotation

We chose a total of 50 images for annotation. This number was based on the availability of our annotator and the time requirements for annotation.

To facilitate comparison with the available root intensity measurements by analysing the same region of the image as [51], the images were cropped from their original dimensions of $4608\times 2592$ pixels to $3991\times 1842$ pixels which corresponds to an area of approximately 300 $\times$ 170 mm of the surface of the rhizotron. This was done by removing the right side of the image where an overlap between images is often present and the top and bottom which included the metal frame around the acrylic glass.

**Fig. 3:**
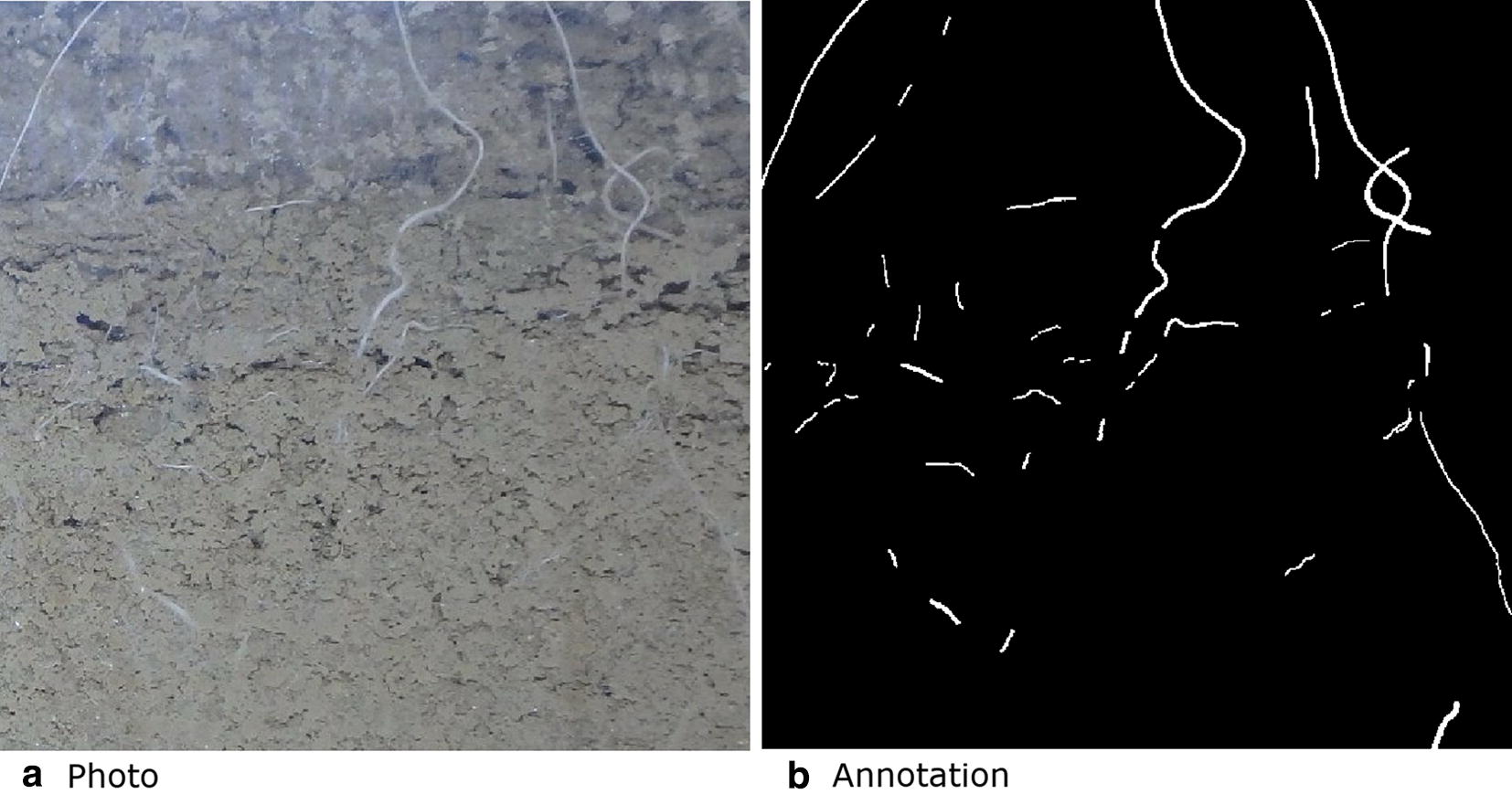
Sub region of one of the photos in the training data. **a** Roots and soil as seen through the transparent acrylic glass on the surface of one of the rhizotrons and **b** is the corresponding annotation showing root pixels in white and all other pixels in black. Annotations like these were used for training the U-Net CNN

A detailed per-pixel annotation (Fig. 3) was then created as a separate layer in Photoshop by a trained agronomist with extensive experience using the line-intersect method. Annotation took approximately 30 min per image with the agronomist labelling all pixels which they perceived to be root.

The number of annotated root pixels ranged from 0 to 203533 (2.8%) per image.

### Data split

During the typical training process of a neural network, the labelled or annotated data is split into a training, validation and test dataset. The training set is used to optimize a neural network using a process called Stochastic Gradient Descent (SGD) where the weights (parameters) are adjusted in such a way that segmentation performance improves. The validation set is used for giving an indication of system performance during the training procedure and tuning the so-called hyper-parameters, not optimised by SGD such as the learning rate. See the section U-Net Implementation for more details. The test set performance is only calculated once after the neural network training process is complete to ensure an unbiased indication of performance.

Firstly, we selected 10 images randomly for the test set. As the test set only contained 10 images, this meant the full range of panel heights could not be included. One image was selected from all panel heights except for 13, 17, 18 and 20. The test set was not viewed or used in the computation of any statistics during the model development process, which means it can be considered as unseen data when evaluating performance. Secondly, from the remaining 40 images we removed two images. One because it didn’t contain any roots and another because a sticker was present on the top of the acrylic. Thirdly, the remaining 38 images were split into split into training and validation datasets.

We used the root pixel count from the annotations to guide the split of the images into a train and validation data-set. The images were ordered by the number of root pixels in each image and then 9 evenly spaced images were selected for the validation set with the rest being assigned to the training set. This was to ensure a range of root intensities was present in both training and validation sets.

### Metrics

To evaluate the performance of the model during development and testing we used $F_1$. We selected $F_1$ as a metric because we were interested in a system which would be just as likely to overestimate as it would underestimate the roots in a given photo. That meant precision and recall were valued equally. In this context precision is the ratio of correctly predicted root pixels to the number of pixels predicted to be root and recall is the ratio of correctly predicted root pixels to the number of actual root pixels in the image. Both recall and precision must be high for $F_1$ to be high.

1 $$\begin{aligned} F_{1} = 2 \cdot {\frac{\text {precision} \cdot \text {recall}}{\text {precision} + \text {recall}}} \end{aligned}$$

The $F_1$ of the segmentation output was calculated using the training and validation sets during system development. The completed system was then evaluated using the test set in order to provide a measure of performance on unseen data. We also report accuracy, defined as the ratio of correctly predicted to total pixels in an image.

In order to facilitate comparison and correlation with line-intersect counts, we used an approach similar to [53] to convert a root segmentation to a length estimate. The scikit-image skeletonize function was used to first thin the segmentation and then the remaining pixels were counted. This approach was used for both the baseline and the U-Net segmentations.

For the test set we also measured correlation between the root length of the output segmentation and the manual root intensity given by the line-intersect method. We also measured correlation between the root length of our manual per-pixel annotations and the U-Net output segmentations for our held out test set. To further quantify the effectiveness of the system as a replacement for the line-intersect method, we obtained the coefficient of determination ($r^2$) for the root length given by our segmentations and root intensity given by the line-intersect method for 867 images. Although line-intersect counts were available for 892 images, 25 images were excluded from our correlation analysis as they had been used in the training dataset.

### Frangi vesselness implementation

For our baseline method we built a system using the Frangi Vesselness enhancement filter [50]. We selected the Frangi filter based on the observation that the roots look similar in structure to blood vessels, for which the Frangi filter was originally designed. We implemented the system using the Python programming language (version 3.6.4), using the scikit-image [54] (version 0.14.0) version of Frangi. Vesselness refers to a measure of tubularity that is predicted by the Frangi filter for a given pixel in the image. To obtain a segmentation using the Frangi filter we thresholded the output so only regions of the image above a certain vesselness level would be classified as roots. To remove noise we further processed the segmentation output using connected component analysis to remove regions less than a threshold of connected pixels. To find optimal parameters for both the thresholds and the parameters for the Frangi filter we used the Covariance Matrix Adaptation Evolution Strategy (CMA-ES) [55]. In our case the objective function to be minimized was $1 - mean(F_1)$ where $mean(F_1)$ is the mean of the $F_1$ scores of the segmentations produced from the thresholded Frangi filter output.

### U-Net implementation

#### Architecture

We implemented a U-Net CNN in Python (version 3.6.4) using PyTorch [56] which is an open source machine learning library which utilizes GPU accelerated tensor operations. PyTorch has convenient utilities for defining and optimizing neural networks. We used an NVIDIA TITAN Xp 12 GB GPU. Except for the input layer which was modified to receive RGB instead of a single channel, our network had the same number of layers and dimensions as the original U-Net [49]. We applied Group norm [57] after all ReLU activations as opposed to Batch norm [58] as batch sizes as small as ours can cause issues due to inaccurate batch statistics degrading the quality of the resulting models [59]. The original U-Net proposed in [49] used Dropout which we avoided as in some cases the combination of dropout and batch normalisation can cause worse results [60]. He initialisation [61] was used for all layers.

#### Instance selection

**Fig. 4:**
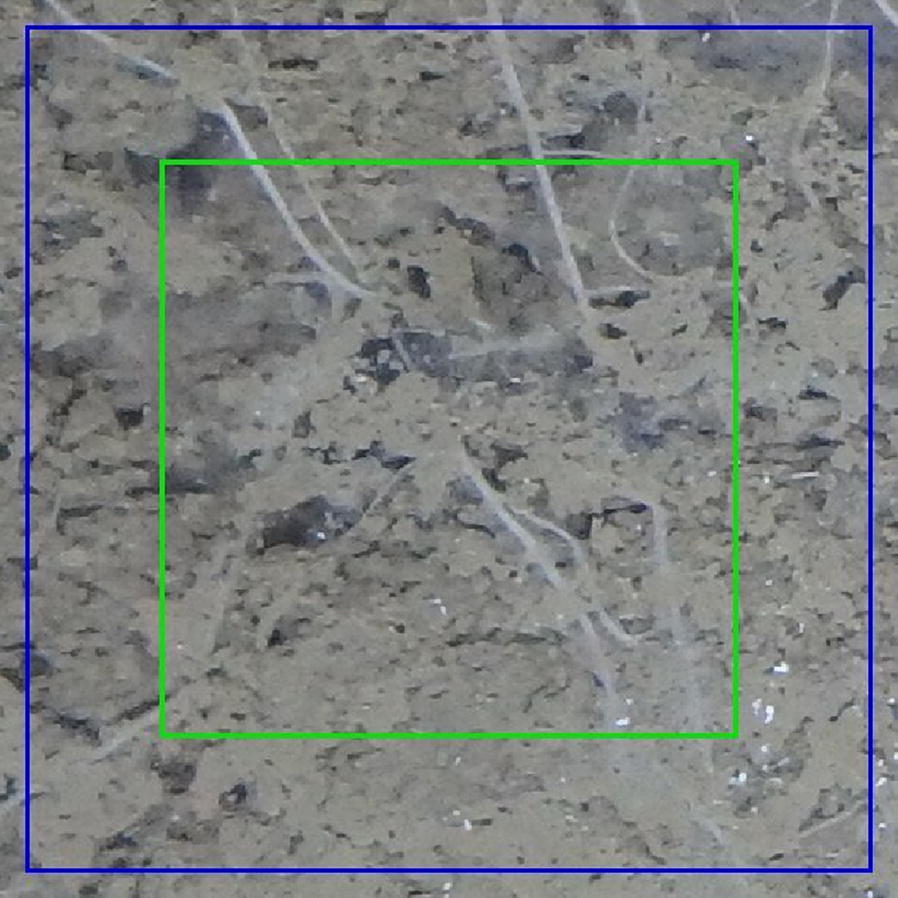
U-Net receptive field input size (blue) and output size (green). The receptive field is the region of the input data which is provided to the neural network. The output size is the region of the original image which the output segmentation is for. The output is smaller than the input to ensure sufficient context for the classification of each pixel in the output

The network takes tiles with size $572 \times 572$ as input and outputs a segmentation for the centre $388 \times 388$ region for each tile (Fig. 4). We used mirroring to pad the full image before extracting tiles. Mirroring in this context means the image was reflected at the edges to make it bigger and provide some synthetic context to allow segmentation at the edges of the image. In neural network training an epoch refers to a full pass over the training data. Typically several epochs are required to reach good performance. At the start of each epoch we extracted 90 tiles with random locations from each of the training images. These tiles were then filtered down to only those containing roots and then a maximum of 40 was taken from what ever was left over. This meant images with many roots would still be limited to 40 tiles. The removal of parts of the image which does not contain roots has similarity to the work of [62] who made the class imbalance problem less severe by cropping regions containing empty space. When training U-Net with mini batch SGD, each item in a batch is an image tile and multiple tiles are input into the network simultaneously. Using tiles as opposed to full images gave us more flexibility during experimentation as we could adjust the batch size depending on the available GPU memory. When training the network we used a batch size of 4 to ensure we did not exceed the limits of the GPU memory. Validation metrics were still calculated using all tiles with and without soil in the validation set.

#### Preprocessing and augmentation

**Fig. 5:**
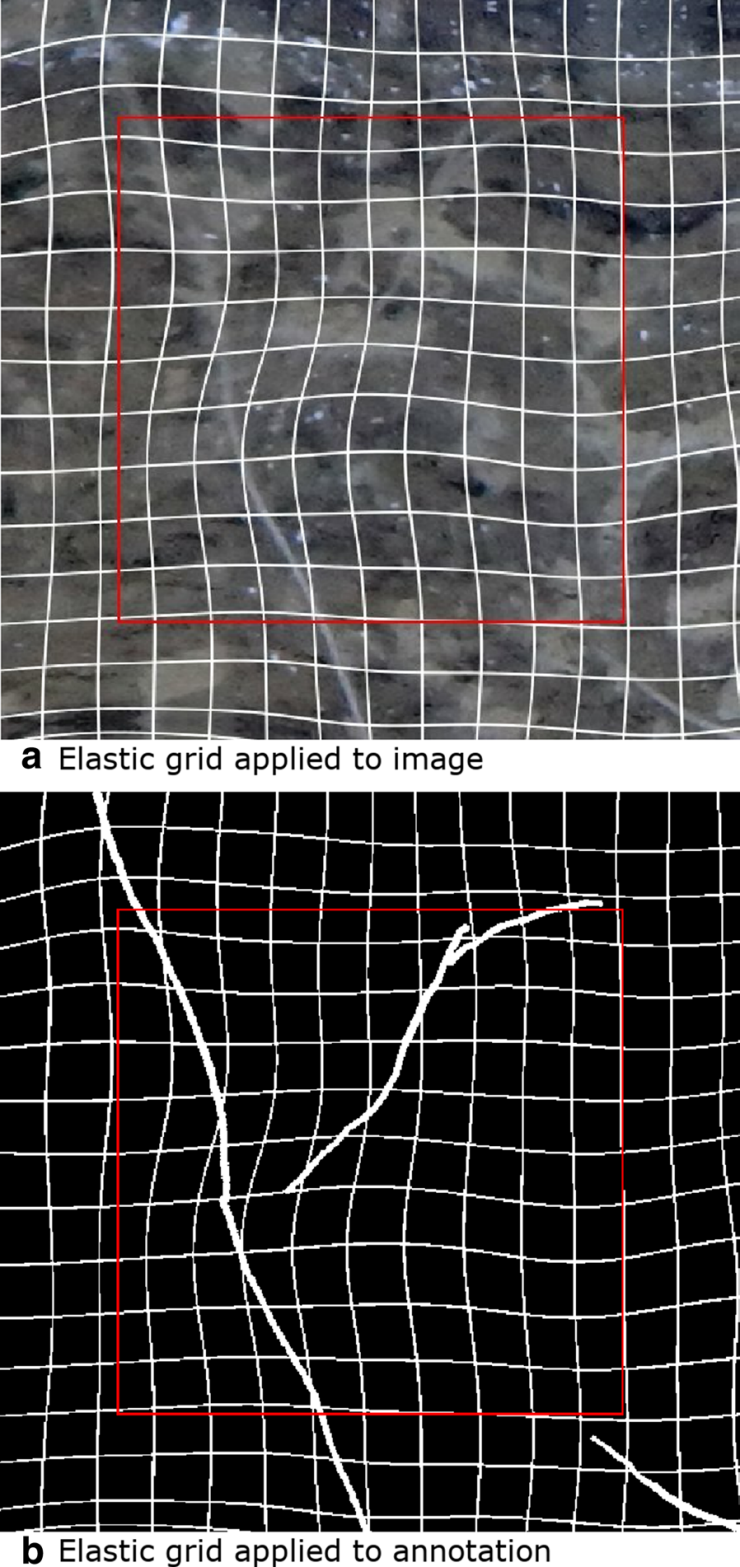
**a** Elastic grid applied to an image tile and **b** corresponding annotation. A white grid is shown to better illustrate the elastic grid effect. A red rectangle illustrates the region which will be segmented. Augmentations such as elastic grid are designed to increase the likelihood that the network will work on similar data that is not included in the training set

Each individual image tile was normalised to $[-0.5, +0.5]$ as centering inputs improves the convergence of networks trained with gradient descent [63]. Data augmentation is a way to artificially expand a dataset and has been found to improve the accuracy of CNNs for image classification [64]. We used color jitter as implemented in PyTorch, with the parameters 0.3, 0.3, 0.2 and 0.001 for brightness, contrast saturation and hue respectively. We implemented elastic grid deformation (Fig. 5) as described by [65] with a probability of 0.9. Elastic grid deformations are parameterized by the standard deviation of a Gaussian distribution $\sigma$ which is an elasticity coefficient and $\alpha$ which controls the intensity of the deformation. As opposed to [65] who suggests a constant value for $\sigma$ and $\alpha$, we used an intermediary parameter $\gamma$ sampled from [0.0, 1.0) uniformly. $\gamma$ was then used as an interpolation co-efficient for both $\sigma$ from [15, 60] and $\alpha$ from [200, 2500]. We found by visual inspection that the appropriate $\alpha$ was larger for a larger $\sigma$. If a too large $\alpha$ was used for a given $\sigma$ then the image would look distorted in unrealistic ways. The joint interpolation of both $\sigma$ and $\alpha$ ensured that the maximum intensity level for a given elasticity coefficient would not lead to over distorted and unrealistic looking deformations. We further scaled $\alpha$ by a random amount from [0.4, 1) so that less extreme deformations would also be applied. We consider the sampling of tiles from random locations within the larger images to provide similar benefits to the commonly used random cropping data augmentation procedure. The augmentations were ran on 8 CPU threads during the training process.

#### Loss

Loss functions quantify our level of unhappiness with the network predictions on the training set [66]. During training the network outputs a predicted segmentation for each input image. The loss function provides a way to measure the difference between the segmentation output by the network and the manual annotations. The result of the loss function is then used to update the network weights in order to improve its performance on the training set. We used the Dice loss as implemented in V-Net [67]. Only 0.54% of the pixels in the training data were roots which represents a class imbalance. Training on imbalanced datasets is challenging because classifiers are typically designed to optimise overall accuracy which can cause minority classes to be ignored [68]. Experiments on CNNs in particular have shown the effect of class imbalance to be detrimental to performance [69] and can cause issues with convergence. The Dice loss is an effective way to handle class imbalanced datasets as errors for the minority class will be given more significance. For predictions *p*, ground truth annotation *g*, and number of pixels in an image *N*, Dice loss was computed as:

2 $$\begin{aligned} DL=1 - \frac{2 (p \cap g)}{p \cup g} =1 - \frac{2\sum _{i}^{N}p_{i}g_{i}}{\sum _{i}^{N}p_{i}+\sum _{i}^{N}g_{i}} \end{aligned}$$

The Dice coefficient corresponds to $F_1$ when there are only two classes and ranges from 0 to 1. It is higher for better segmentations. Thus it is subtracted from 1 to convert it to a loss function to be minimized. We combined the Dice loss with cross-entropy multiplied by 0.3, which was found using trial and error. This combination of loss functions was used because it provided better results than either loss function in isolation during our preliminary experiments.

#### Optimization

**Fig. 6:**
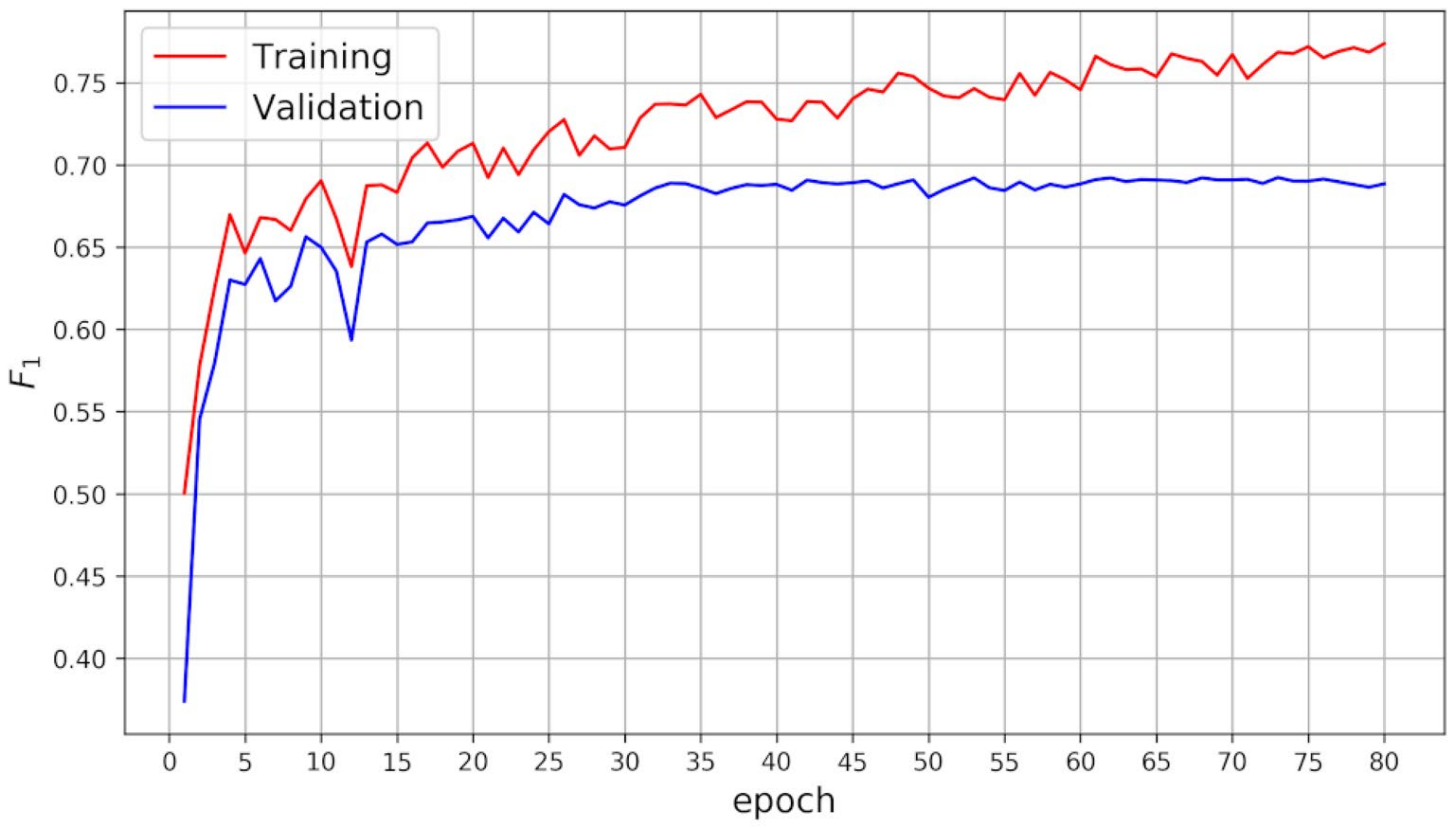
$F_1$ on training and validation data sets. $F_1$ is a measure of the system accuracy. The training $F_1$ continues to improve whilst the validation $F_1$ appears to plateau at around epoch 40. This is because the network is starting to fit to noise and other anomalies in the training data which are not present in the validation images

We used SGD with Nesterov momentum based on the formula from [70]. We used a value of 0.99 for momentum as this was used in the original U-Net implementation. We used an initial learning rate of 0.01 which was found by using trial and error whilst monitoring the validation and training $F_1$. The learning rate alters the magnitude of the updates to the network weights during each iteration of the training procedure. We used weight decay with a value of $1 \times 10^{-5}$. A learning rate schedule was used where the learning rate would be multiplied by 0.3 every 30 epochs. Adaptive optimization methods such as Adam [71] were avoided due to results showing they can cause worse generalisation behaviour [72, 73]. The $F_1$ computed on both the augmented training and validation after each epoch is shown in Fig. 6.

## Results

We succeeded in getting both the U-Net and the Frangi filter system to segment roots in the images in the train and validation datasets (Table 2) as well as the held out test set (Table 3). As $F_1$, recall and precision is not defined for images without roots we report the results on all images combined (Table 3). We report the mean and standard deviation of the per image results from the images which contain roots (Table 4). When computing these per image statistics we can see that U-Net performed better than the Frangi system for all metrics attained.

**Table 2:** Best U-Net model results on the train set and the validation set used for early stopping

	Training	Validation
Accuracy	0.996	0.996
Precision	0.758	0.674
Recall	0.780	0.712
$F_1$	0.769	0.692

These train set results are calculated on data affected by both instance selection and augmentation

**Table 3:** Metrics on all images combined for the held out test set for the Frangi and U-Net segmentation systems

	Frangi	U-Net
Accuracy	0.996	0.997
$F_1$	0.462	0.701
Precision	0.660	0.659
Recall	0.355	0.748
Prediction mean	0.002	0.006
True mean	0.005	0.005

**Table 4:** Mean and standard deviation of results on images containing roots

	Frangi	U-Net
$F_1$ mean	0.463	0.705
$F_1$ standard deviation	0.085	0.040
Recall mean	0.361	0.749
Recall standard deviation	0.081	0.042
Precision mean	0.660	0.666
Precision standard deviation	0.087	0.043

These are computed by taking the mean of the metrics computed on each of the 8 images containing roots. The 2 images without roots are excluded as for these $F_1$, precision and recall are undefined

### Train and validation set metrics

The final model parameters were selected based on the performance on the validation set. The best validation results were attained after epoch 73 after approximately 9 h and 34 min of training. The performance on the training set was higher than the validation set (Table 2). As parameters have been adjusted based on the data in the training and validation datasets these results are unlikely to be reliable indications of the model performance on new data so we report the performance on an unseen test set in the next section.

### Test set results

**Fig. 7:**
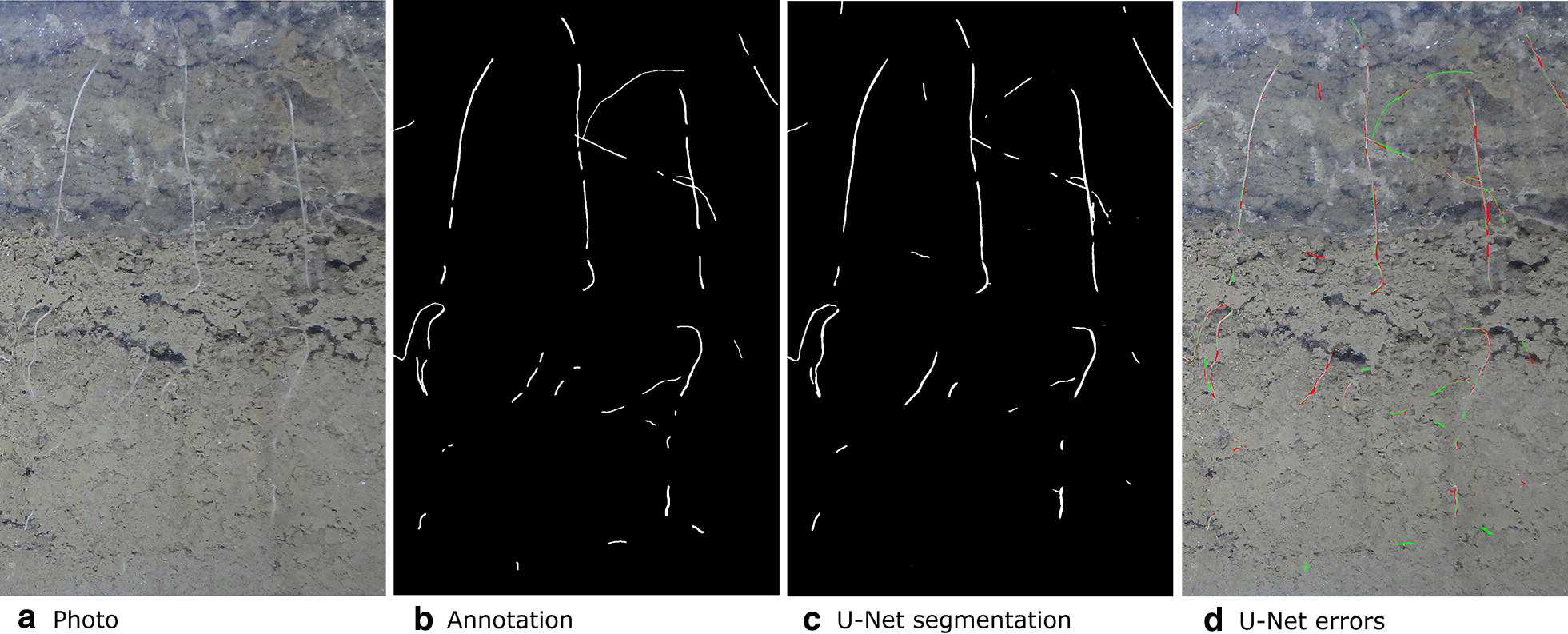
Original photo, annotation, segmentation output from U-Net and errors. To illustrate the errors the false positives are shown in red and the false negatives are shown in green. This image is a subregion of a larger image for which U-Net got the worst (lowest) $F_1$

**Fig. 8:**
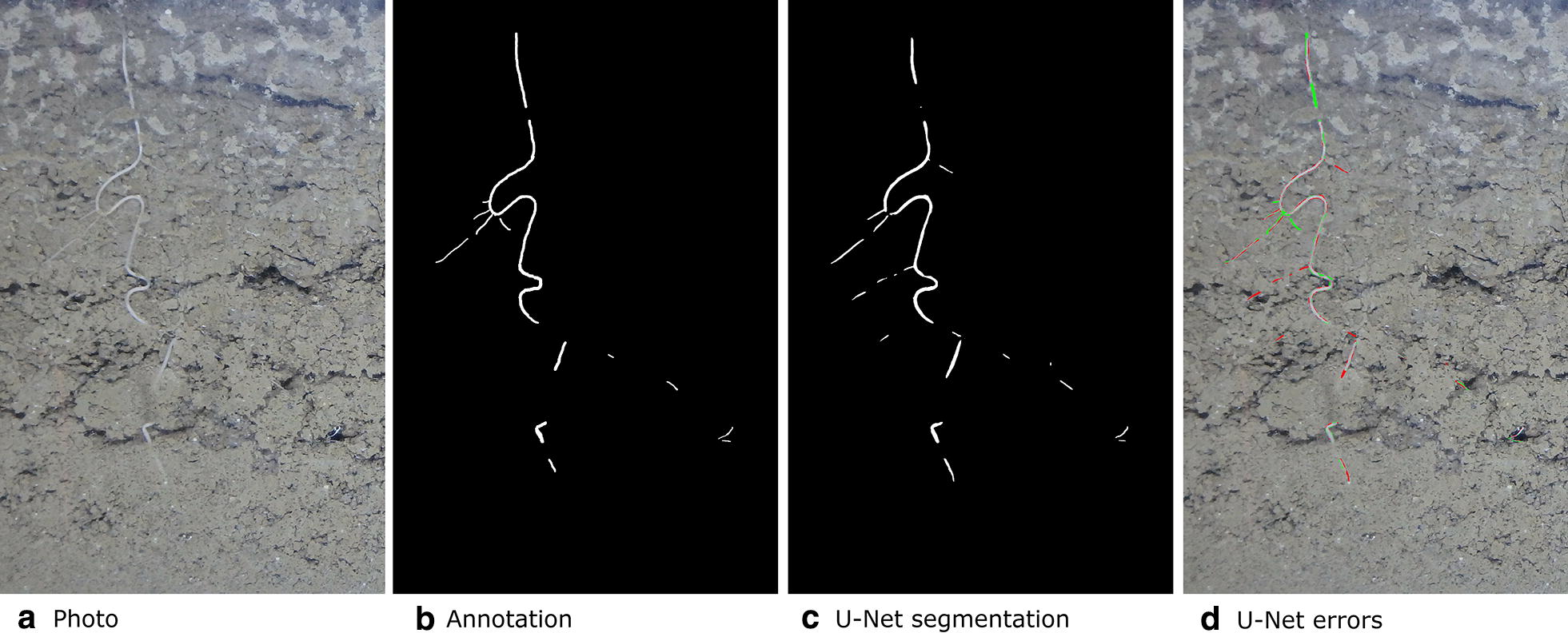
Original photo, annotation, segmentation output from U-Net and errors. To illustrate the errors the false positives are shown in red and the false negatives are shown in green. This image is a subregion of a larger image for which U-Net got the best (highest) $F_1$. The segmentation also contains roots which were missed by the annotator. We were able to confirm this by having the annotator review these particular errors

**Fig. 9:**
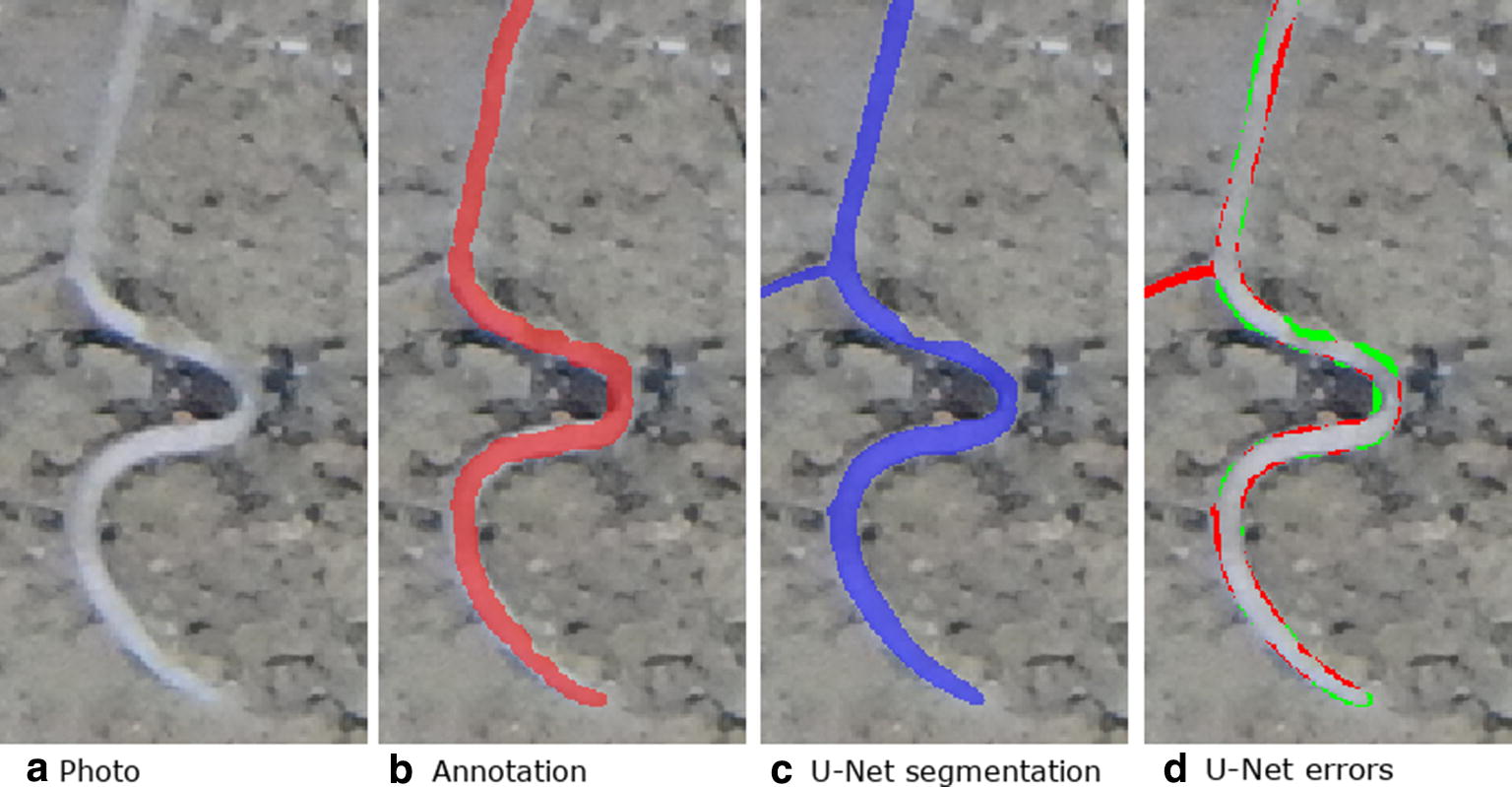
From left to right: Image, annotation overlaid over image in red, U-Net segmentation overlaid over image in blue, errors with false positive shown in red and false negative shown in green. Many of the errors are along an ambiguous boundary region between the root and soil. Much of the error region is caused by annotation, rather than CNN segmentation errors

The overall percentage of root pixels in the test data was 0.49%, which is lower than either the training or validation dataset. Even on the image with the highest errors the CNN is able to predict many of the roots correctly (Fig. 7). Many of the errors appear to be on the root boundaries. Some of the fainter roots are also missed by the CNN. For the image with the highest (best) $F_1$ the U-Net segmentation appears very similar to the original annotation (Fig. 8). The segmentation also contains roots which where missed by the annotator (Fig. 8d) which we were able to confirm by asking the annotator to review the results. U-Net was also often able to segment the root-soil boundary more cleanly than the annotator (Fig. 9). False negatives can be seen at the top of the image where the CNN has failed to detect a small section of root (Fig. 8d).

**Fig. 10:**
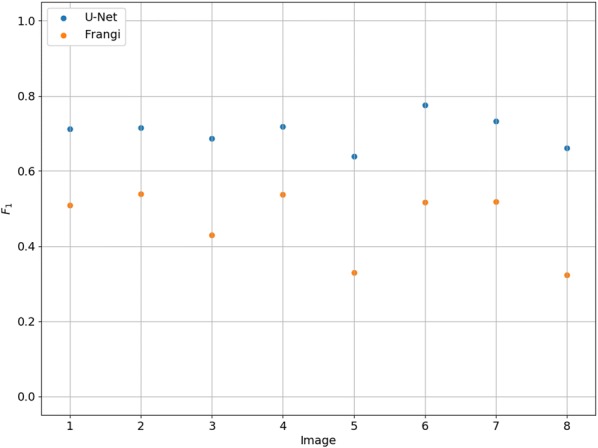
The $F_1$ for the 8 images containing roots for both the Frangi and U-Net systems

The performance of U-Net as measured by $F_1$ was better than that of the Frangi system when computing metrics on all images combined (Table 3). It also had a closer balance between precision and recall. The U-Net segmentations have a higher $F_1$ for all images with roots in the test data (Fig. 10). Some segmentations from the Frangi system have an $F_1$ below 0.4 whilst all the U-Net segmentations give an $F_1$ above 0.6 with the highest being just less than 0.8. The average predicted value for U-Net was over twice that of the Frangi system. This means U-Net predicted twice as many pixels to be root as Frangi did.

The slight over estimation of total root pixels explains why recall is higher than precision for U-Net. The accuracy is above 99% for both systems. This is because accuracy is measured as the ratio of pixels predicted correctly and the vast majority of pixels are soil that both systems predicted correctly.

**Fig. 11:**
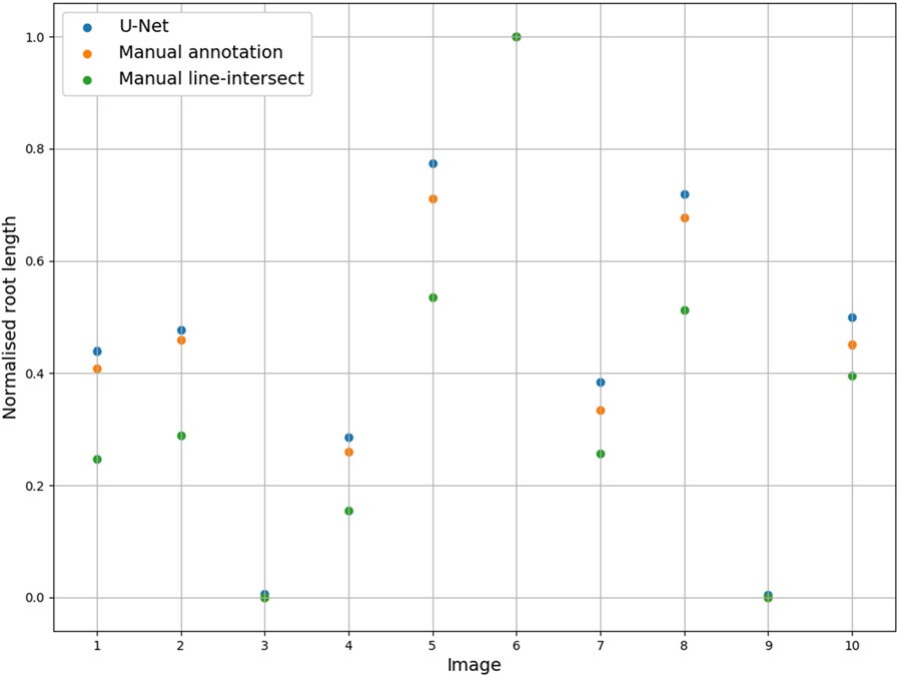
Normalised root length from the U-Net segmentations, manual annotations and the line-intersect counts for the 10 test images. The measurements are normalised using the maximum value. All three methods have the same maximum value (Image 6)

For the two images which did not contain roots each misclassified pixel is counted as a false positive. The Frangi system gave 1997 and 1432 false positives on these images and the U-Net system gave 508 and 345 false positives. The Spearman rank correlation for the corresponding U-Net and line-intersect root intensities for the test data is 0.9848 ($p=2.288 \times 10^{-7}$). The U-Net segmentation can be seen to give a similar root intensity to the manual annotations (Fig. 11).

**Fig. 12:**
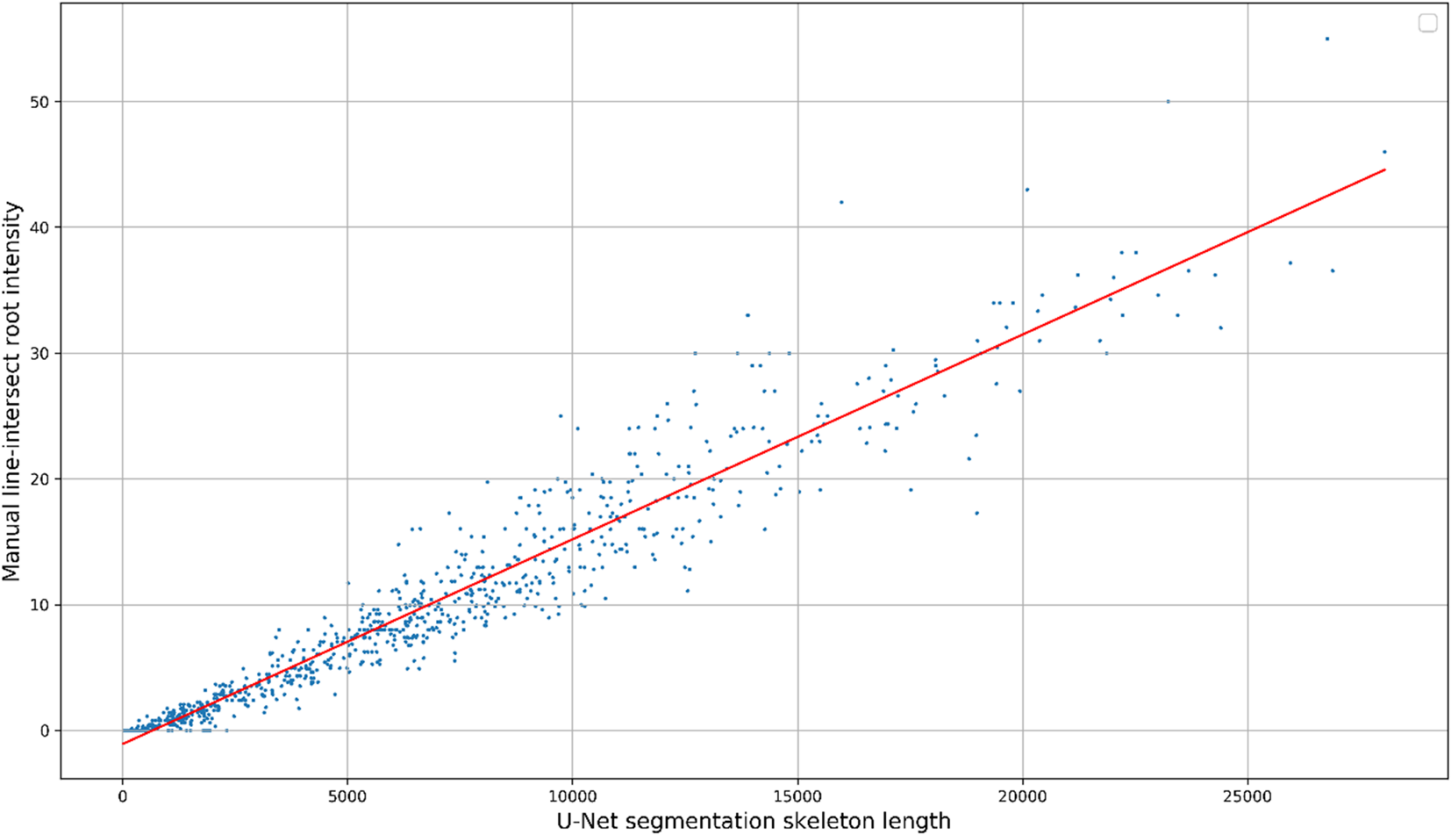
RI vs segmented root length for 867 images taken in 2016. The two measurements have a Spearman rank correlation of 0.9748 and an $R^2$ of 0.9217

We report the root intensity with the segmented root length for 867 images taken in 2016 (Fig. 12). The two measurements have a Spearman rank correlation of 0.9748 $(p < 10^{-8})$ and an $r^2$ of 0.9217. Although the two measurements correlate strongly, there are some notable deviations including images for which U-Net predicted roots not observed by the manual annotator. From this scatter plot we can see that the data is heteroscedastic, forming a cone shape around the regression line with the variance increasing as root intensity increases in both measurements.

## Conclusions

We have demonstrated the feasibility of a U-Net based CNN system for segmenting images of roots in soil and for replacing the manual line-intersect method. The success of our approach is also a demonstration of the feasibility of deep learning in practice for small research groups needing to create their own custom labelled dataset from scratch.

## Discussion

We have presented a method to segment roots from soil using a CNN. The segmentation quality as shown in Figs. 7c and 8c and the approximation of the root length given by our automated method and the manual line-intersect method for the corresponding images as shown in Figs. 11 and 12 are a strong indication that the system works well for the intended task of quantifying roots.

The high correlation coefficient between the measurements from the automated and manual methods supports our hypothesis that a trained U-Net is able to effectively discriminate between roots and soil in RGB photographs. The consistently superior performance of the U-Net system on the unseen test set over the Frangi system as measured by $F_1$ score supports our second hypothesis that a trained U-Net will outperform a Frangi filter based approach.

The good generalisation behaviour and the success of the validation set at closely approximating the test set error indicate we would likely not need as many annotations for validation on future root datasets. As shown in Fig. 12 there are some images for which U-Net predicted roots and the line-intersection count was 0. When investigating these cases we found some false positives caused by scratches in the acrylic glass. Such errors could be problematic as they make it hard to attain accurate estimates of maximum rooting depth as the scratches could cause rooting depth to be overestimated. One way to fix this would be to manually design a dataset with more scratched panels in it in order to train U-Net not to classify them as roots. Another possible approach would be to automatically find difficult regions of images using an active learning approach such as [74] which would allow the network to query which areas of images should be annotated based on its uncertainty.

An oft-stated limitation of CNNs is that they require large scale datasets [75] with thousands of densely labelled images [76] for annotation. In this study we were able to train from scratch, validate and test a CNN with only 50 images which were annotated in a few days by a single agronomist with no annotation or machine learning experience. Our system was also designed to work with an existing photography setup using an ordinary off-the-shelf RGB camera. This makes our method more broadly accessible than methods which require a more complex multi-spectral camera system.

We used a loss function which combined Dice and cross entropy. In preliminary experiments we found this combined loss function to be more effective than either Dice or cross entropy used in isolation. Both [77] and [78] found empirically that a combination of Dice and cross entropy was effective at improving accuracy. Although [77] claims the combination of the loss functions is a way to yield better performance in terms of both pixel accuracy and segmentation metrics, we feel more research is needed to understand the exact benefits of such combined loss functions.

Converting from segmentation to root length was not the focus of the current study. The method we used consisted of skeletonization and then pixel counting. One limitation of this method is that it may lead to different length estimates depending on the orientation of the roots [79]. See [79] for an in depth investigation and proposed solutions.

Finding ways to improve annotation quality would also be a promising direction for further work. Figure 9 shows how even a high quality segmentation will still have a large number of errors due to issues with annotation quality. This makes the $F_1$ given for a segmentation to not be representative of the systems’ true performance. [80] found significant disagreement between human raters in segmenting tumour regions with Dice (equivalent to our $F_1$) scores between 74 and 85%. We suspect a similar level of error is present in our root annotations and that improving annotation quality would improve the metrics. Improved annotation quality would be particularly useful for the test and validation datasets as it would allow us to train the model to a higher performance.

One way to improve the quality of annotations would be to combine various annotations by different experts using a majority vote algorithm such as the one used by [80] although caution should be taken when implementing such methods as in some cases they can accentuate more obvious features, causing an overestimation of performance [81].

It may also be worth investigating ways to reduce the weight of errors very close to the border of an annotation, as seen in Fig. 9, these are often issues with annotation quality or merely ambiguous boundary regions where a labelling of either root or soil should not be detrimental to the $F_1$. One way to solve the problem with misleading errors caused by ambiguous boundary regions is the approach taken by [41] which involved having a boundary region around each area of interest where a classification either way will not affect the overall performance metrics.

We excluded an image not containing roots and an image containing a sticker from our training and validation data. During training we also excluded parts of the image where no roots were found in order to handle the severe class imbalance present in the dataset. A limitation of this approach is that it may be useful for the network to learn to deal with stickers and in some cases, images without roots could contain hard negative examples which the network must learn to handle in order for it to achieve acceptable performance.

For future research we aim to explore how well the segmentation system performance will transfer to photographs from both other crop species and different experimental setups. In our work so far we have explored ways to deal with a limited dataset by using data augmentation. Transfer learning is another technique which has been found to improve the performance of CNNs when compared to training from scratch for small datasets [47]. We can simultaneously investigate both transfer learning and the feasibility of our system to work with different kinds of plants by fine-tuning our existing network on root images from new plant species. [82] found pre-training U-Net to both substantially reduce training time and prevent overfitting. Interestingly, they pre-trained U-Net on two different datasets containing different types of images and found similar performance improvements in both cases. Such results indicate that pre-training U-Net using images which are substantially different from our root images may also provide performance advantages. Contra to this, [83] found training from scratch to give equivalent results to a transfer learning approach, which suggests that in some cases training time rather than final model performance will be the benefit of a transfer learning approach. As shown in Fig. 7, the CNN would leave gaps when a root was covered by large amounts of soil. An approach such as [84] could be used to recover such gaps which may improve the biological relevance of our root length estimates and potentially facilitate the extraction of more detailed root architecture information.

As opposed to U-Net, the Frangi filter is included in popular image processing packages such as MATLAB and scikit-image. Although the Frangi filter was initially simple to implement, we found the scikit-image implementation too slow to facilitate optimisation on our dataset and substantial modifications were required to make optimisation feasible.

Another disadvantage of the CNN we implemented is that as opposed to the Frangi filter, it requires a GPU for training. It is, however, possible to use a CPU for inference. [85] demonstrated that in some cases U-Net can be compressed to 0.1% of it’s original parameter count with a very small drop in accuracy. Such an approach could be useful for making our proposed system more accessible to hardware constrained researchers.

## Data Availability

The data used for the current study is available from [86]. The source code for the systems presented is available from [87] and the trained U-Net model is available from [88].
